# Obituary

**Published:** 1907-06-15

**Authors:** 


### OBITUARY.

Dr. William Newton Tuller, 24 years old, son of Dr. R. B. Tuller, of Chicago, died on Wednesday at Carlsbad, N. M., from typhoid fever contracted as he was recovering from an operation for appendicitis. He had gone west a year ago on account of ill health. He was born and educated in Chicago, and was a graduate of the Chicago College of Dental Surgery, of which institution his father has been a member of the faculty for ten years. We extend our cordial sympathy to Dr. Tuller, whose loss in the death of his beloved son is so severe. He was a young man of great promise, and had his health been equal to his spirit and ambition, he would have made his mark in the profession to which he had dedicated himself.

